# Supplementary material for: Heparin-based hydrogel scaffolding alters the transcriptomic profile and increases the chemoresistance of MDA-MB-231 triple-negative breast cancer cells
Source: Biomater Sci. 2020 Feb 13;8(10):2786–96. doi: 10.1039/c9bm01481k (PMC7497406; doi:10.1039/c9bm01481k)
Supplement: Supplementary file 2 [file BM-008-C9BM01481K-s002.zip › Supplementary File 4/HP-BhydrogelvControl/Pathways/my_analysis.Gsea.1545201079907/HALLMARK_TNFA_SIGNALING_VIA_NFKB.html]

Details for gene set HALLMARK\_TNFA\_SIGNALING\_VIA\_NFKB[GSEA]

|  || Dataset | expr.class.cls#HYD\_versus\_CONTROL.class.cls#HYD\_versus\_CONTROL\_repos |
| Phenotype | class.cls#HYD\_versus\_CONTROL\_repos |
| Upregulated in class | HYD |
| GeneSet | HALLMARK\_TNFA\_SIGNALING\_VIA\_NFKB |
| Enrichment Score (ES) | 0.2222426 |
| Normalized Enrichment Score (NES) | 0.9081915 |
| Nominal p-value | 0.68053854 |
| FDR q-value | 0.8936127 |
| FWER p-Value | 1.0 |
Table: GSEA Results Summary

  

Fig 1: Enrichment plot: HALLMARK\_TNFA\_SIGNALING\_VIA\_NFKB      
 Profile of the Running ES Score & Positions of GeneSet Members on the Rank Ordered List

  

| PROBE | DESCRIPTION (from dataset) | GENE SYMBOL | GENE\_TITLE | RANK IN GENE LIST | RANK METRIC SCORE | RUNNING ES | CORE ENRICHMENT || 1 | MAFF | na |  |  | 475 | 2.376 | -0.0030 | Yes |
| 2 | IER5 | na |  |  | 719 | 2.098 | 0.0036 | Yes |
| 3 | MAP3K8 | na |  |  | 1059 | 1.833 | 0.0027 | Yes |
| 4 | SIK1 | na |  |  | 1086 | 1.811 | 0.0181 | Yes |
| 5 | DUSP1 | na |  |  | 1364 | 1.645 | 0.0187 | Yes |
| 6 | ID2 | na |  |  | 1383 | 1.637 | 0.0329 | Yes |
| 7 | PPP1R15A | na |  |  | 1561 | 1.546 | 0.0379 | Yes |
| 8 | EHD1 | na |  |  | 1687 | 1.495 | 0.0451 | Yes |
| 9 | TNIP2 | na |  |  | 1779 | 1.452 | 0.0538 | Yes |
| 10 | IRS2 | na |  |  | 1821 | 1.435 | 0.0649 | Yes |
| 11 | STAT5A | na |  |  | 1889 | 1.409 | 0.0744 | Yes |
| 12 | PTGER4 | na |  |  | 1903 | 1.406 | 0.0867 | Yes |
| 13 | FOSB | na |  |  | 1957 | 1.379 | 0.0966 | Yes |
| 14 | GADD45B | na |  |  | 1972 | 1.375 | 0.1086 | Yes |
| 15 | JUN | na |  |  | 1992 | 1.366 | 0.1202 | Yes |
| 16 | GADD45A | na |  |  | 2424 | 1.200 | 0.1086 | Yes |
| 17 | ATF3 | na |  |  | 2583 | 1.150 | 0.1110 | Yes |
| 18 | MAP2K3 | na |  |  | 2628 | 1.135 | 0.1191 | Yes |
| 19 | TRIP10 | na |  |  | 2676 | 1.124 | 0.1271 | Yes |
| 20 | NR4A1 | na |  |  | 2699 | 1.116 | 0.1362 | Yes |
| 21 | TNFSF9 | na |  |  | 2848 | 1.072 | 0.1383 | Yes |
| 22 | SMAD3 | na |  |  | 2896 | 1.064 | 0.1457 | Yes |
| 23 | HES1 | na |  |  | 3253 | 0.976 | 0.1360 | Yes |
| 24 | SPHK1 | na |  |  | 3452 | 0.927 | 0.1342 | Yes |
| 25 | NFKB1 | na |  |  | 3668 | 0.878 | 0.1310 | Yes |
| 26 | FOSL1 | na |  |  | 3713 | 0.868 | 0.1367 | Yes |
| 27 | DENND5A | na |  |  | 3761 | 0.856 | 0.1421 | Yes |
| 28 | CLCF1 | na |  |  | 3765 | 0.856 | 0.1499 | Yes |
| 29 | MYC | na |  |  | 3771 | 0.854 | 0.1575 | Yes |
| 30 | PER1 | na |  |  | 3822 | 0.846 | 0.1627 | Yes |
| 31 | SLC2A6 | na |  |  | 3851 | 0.842 | 0.1690 | Yes |
| 32 | CCNL1 | na |  |  | 3919 | 0.828 | 0.1731 | Yes |
| 33 | FJX1 | na |  |  | 3977 | 0.816 | 0.1777 | Yes |
| 34 | NR4A3 | na |  |  | 3995 | 0.813 | 0.1843 | Yes |
| 35 | PMEPA1 | na |  |  | 4320 | 0.748 | 0.1742 | Yes |
| 36 | CD83 | na |  |  | 4324 | 0.747 | 0.1809 | Yes |
| 37 | PLK2 | na |  |  | 4474 | 0.721 | 0.1797 | Yes |
| 38 | ZBTB10 | na |  |  | 4606 | 0.692 | 0.1792 | Yes |
| 39 | SOCS3 | na |  |  | 4653 | 0.684 | 0.1832 | Yes |
| 40 | KDM6B | na |  |  | 4705 | 0.675 | 0.1867 | Yes |
| 41 | ICOSLG | na |  |  | 4783 | 0.662 | 0.1888 | Yes |
| 42 | MARCKS | na |  |  | 4831 | 0.652 | 0.1923 | Yes |
| 43 | EDN1 | na |  |  | 4838 | 0.651 | 0.1980 | Yes |
| 44 | BMP2 | na |  |  | 4891 | 0.643 | 0.2012 | Yes |
| 45 | CFLAR | na |  |  | 4965 | 0.633 | 0.2033 | Yes |
| 46 | SERPINB2 | na |  |  | 4996 | 0.627 | 0.2075 | Yes |
| 47 | LIF | na |  |  | 5094 | 0.614 | 0.2080 | Yes |
| 48 | LDLR | na |  |  | 5135 | 0.608 | 0.2116 | Yes |
| 49 | RNF19B | na |  |  | 5374 | 0.573 | 0.2043 | Yes |
| 50 | NFAT5 | na |  |  | 5394 | 0.571 | 0.2086 | Yes |
| 51 | TIPARP | na |  |  | 5438 | 0.563 | 0.2116 | Yes |
| 52 | HBEGF | na |  |  | 5567 | 0.542 | 0.2098 | Yes |
| 53 | FUT4 | na |  |  | 5570 | 0.541 | 0.2147 | Yes |
| 54 | IRF1 | na |  |  | 5648 | 0.533 | 0.2156 | Yes |
| 55 | RELB | na |  |  | 5835 | 0.509 | 0.2105 | Yes |
| 56 | EGR1 | na |  |  | 5934 | 0.496 | 0.2100 | Yes |
| 57 | PANX1 | na |  |  | 5941 | 0.495 | 0.2142 | Yes |
| 58 | EGR3 | na |  |  | 6013 | 0.483 | 0.2149 | Yes |
| 59 | GCH1 | na |  |  | 6157 | 0.463 | 0.2117 | Yes |
| 60 | ETS2 | na |  |  | 6164 | 0.462 | 0.2156 | Yes |
| 61 | FOS | na |  |  | 6167 | 0.461 | 0.2198 | Yes |
| 62 | NFKB2 | na |  |  | 6204 | 0.458 | 0.2221 | Yes |
| 63 | PTGS2 | na |  |  | 6325 | 0.440 | 0.2199 | Yes |
| 64 | PTX3 | na |  |  | 6358 | 0.435 | 0.2222 | Yes |
| 65 | CEBPB | na |  |  | 6839 | 0.379 | 0.2005 | No |
| 66 | CXCL10 | na |  |  | 6890 | 0.372 | 0.2013 | No |
| 67 | TNFAIP3 | na |  |  | 6980 | 0.364 | 0.2000 | No |
| 68 | SQSTM1 | na |  |  | 7012 | 0.359 | 0.2017 | No |
| 69 | IL18 | na |  |  | 7027 | 0.356 | 0.2042 | No |
| 70 | CXCL3 | na |  |  | 7031 | 0.355 | 0.2074 | No |
| 71 | KLF4 | na |  |  | 7185 | 0.341 | 0.2025 | No |
| 72 | IL6 | na |  |  | 7244 | 0.333 | 0.2025 | No |
| 73 | TNF | na |  |  | 7258 | 0.331 | 0.2049 | No |
| 74 | CXCL2 | na |  |  | 7437 | 0.314 | 0.1984 | No |
| 75 | ZC3H12A | na |  |  | 7464 | 0.311 | 0.1999 | No |
| 76 | CCND1 | na |  |  | 7494 | 0.308 | 0.2012 | No |
| 77 | TNIP1 | na |  |  | 7539 | 0.300 | 0.2017 | No |
| 78 | FOSL2 | na |  |  | 7639 | 0.286 | 0.1991 | No |
| 79 | BTG2 | na |  |  | 7702 | 0.284 | 0.1985 | No |
| 80 | MCL1 | na |  |  | 7729 | 0.284 | 0.1998 | No |
| 81 | KLF2 | na |  |  | 7772 | 0.276 | 0.2001 | No |
| 82 | CXCL1 | na |  |  | 7814 | 0.270 | 0.2005 | No |
| 83 | TRAF1 | na |  |  | 8359 | 0.212 | 0.1738 | No |
| 84 | LAMB3 | na |  |  | 8610 | 0.191 | 0.1624 | No |
| 85 | IL23A | na |  |  | 8621 | 0.190 | 0.1637 | No |
| 86 | NFKBIE | na |  |  | 8778 | 0.170 | 0.1570 | No |
| 87 | PFKFB3 | na |  |  | 8864 | 0.157 | 0.1540 | No |
| 88 | TNC | na |  |  | 9035 | 0.149 | 0.1465 | No |
| 89 | F3 | na |  |  | 9090 | 0.142 | 0.1449 | No |
| 90 | SGK1 | na |  |  | 9100 | 0.141 | 0.1458 | No |
| 91 | KYNU | na |  |  | 9128 | 0.137 | 0.1456 | No |
| 92 | IL1B | na |  |  | 9136 | 0.136 | 0.1465 | No |
| 93 | RHOB | na |  |  | 9250 | 0.124 | 0.1417 | No |
| 94 | NR4A2 | na |  |  | 9329 | 0.112 | 0.1386 | No |
| 95 | IL1A | na |  |  | 9651 | 0.080 | 0.1225 | No |
| 96 | RCAN1 | na |  |  | 9664 | 0.078 | 0.1226 | No |
| 97 | CEBPD | na |  |  | 9727 | 0.072 | 0.1200 | No |
| 98 | PTPRE | na |  |  | 9844 | 0.059 | 0.1144 | No |
| 99 | IL15RA | na |  |  | 9909 | 0.053 | 0.1116 | No |
| 100 | ICAM1 | na |  |  | 9949 | 0.046 | 0.1099 | No |
| 101 | ABCA1 | na |  |  | 10121 | 0.026 | 0.1012 | No |
| 102 | BCL6 | na |  |  | 10150 | 0.022 | 0.0999 | No |
| 103 | EGR2 | na |  |  | 10492 | -0.004 | 0.0820 | No |
| 104 | BCL3 | na |  |  | 10566 | -0.012 | 0.0783 | No |
| 105 | NINJ1 | na |  |  | 10608 | -0.017 | 0.0763 | No |
| 106 | ZFP36 | na |  |  | 10619 | -0.019 | 0.0759 | No |
| 107 | TAP1 | na |  |  | 10646 | -0.022 | 0.0748 | No |
| 108 | TGIF1 | na |  |  | 10654 | -0.024 | 0.0746 | No |
| 109 | DUSP5 | na |  |  | 10948 | -0.059 | 0.0598 | No |
| 110 | IER2 | na |  |  | 11016 | -0.068 | 0.0569 | No |
| 111 | JUNB | na |  |  | 11042 | -0.071 | 0.0562 | No |
| 112 | CD44 | na |  |  | 11081 | -0.075 | 0.0549 | No |
| 113 | KLF10 | na |  |  | 11366 | -0.099 | 0.0409 | No |
| 114 | PLAUR | na |  |  | 11509 | -0.112 | 0.0345 | No |
| 115 | PLAU | na |  |  | 11513 | -0.113 | 0.0354 | No |
| 116 | YRDC | na |  |  | 11535 | -0.117 | 0.0353 | No |
| 117 | CDKN1A | na |  |  | 11746 | -0.145 | 0.0256 | No |
| 118 | G0S2 | na |  |  | 11856 | -0.159 | 0.0214 | No |
| 119 | REL | na |  |  | 12166 | -0.195 | 0.0069 | No |
| 120 | TNFRSF9 | na |  |  | 12175 | -0.197 | 0.0083 | No |
| 121 | NFKBIA | na |  |  | 12331 | -0.218 | 0.0022 | No |
| 122 | CCL2 | na |  |  | 12375 | -0.222 | 0.0020 | No |
| 123 | DUSP4 | na |  |  | 12770 | -0.255 | -0.0164 | No |
| 124 | CCL20 | na |  |  | 12777 | -0.255 | -0.0143 | No |
| 125 | TLR2 | na |  |  | 12836 | -0.262 | -0.0150 | No |
| 126 | KLF6 | na |  |  | 12855 | -0.265 | -0.0135 | No |
| 127 | SNN | na |  |  | 12897 | -0.271 | -0.0131 | No |
| 128 | PHLDA2 | na |  |  | 12946 | -0.277 | -0.0131 | No |
| 129 | EFNA1 | na |  |  | 13011 | -0.285 | -0.0138 | No |
| 130 | BIRC2 | na |  |  | 13013 | -0.285 | -0.0112 | No |
| 131 | CSF2 | na |  |  | 13029 | -0.288 | -0.0093 | No |
| 132 | CXCL11 | na |  |  | 13037 | -0.289 | -0.0070 | No |
| 133 | RELA | na |  |  | 13082 | -0.296 | -0.0066 | No |
| 134 | DRAM1 | na |  |  | 13242 | -0.315 | -0.0121 | No |
| 135 | IFIH1 | na |  |  | 13280 | -0.322 | -0.0110 | No |
| 136 | SPSB1 | na |  |  | 13363 | -0.332 | -0.0123 | No |
| 137 | GFPT2 | na |  |  | 13386 | -0.335 | -0.0103 | No |
| 138 | B4GALT1 | na |  |  | 13415 | -0.340 | -0.0087 | No |
| 139 | SDC4 | na |  |  | 13470 | -0.349 | -0.0083 | No |
| 140 | MXD1 | na |  |  | 13509 | -0.354 | -0.0070 | No |
| 141 | BHLHE40 | na |  |  | 13518 | -0.356 | -0.0042 | No |
| 142 | INHBA | na |  |  | 13523 | -0.357 | -0.0011 | No |
| 143 | KLF9 | na |  |  | 13547 | -0.359 | 0.0010 | No |
| 144 | SAT1 | na |  |  | 13558 | -0.362 | 0.0039 | No |
| 145 | SOD2 | na |  |  | 13651 | -0.378 | 0.0025 | No |
| 146 | ATP2B1 | na |  |  | 14037 | -0.422 | -0.0138 | No |
| 147 | TUBB2A | na |  |  | 14107 | -0.429 | -0.0135 | No |
| 148 | TRIB1 | na |  |  | 14135 | -0.432 | -0.0109 | No |
| 149 | SERPINB8 | na |  |  | 14190 | -0.440 | -0.0097 | No |
| 150 | TSC22D1 | na |  |  | 14324 | -0.468 | -0.0124 | No |
| 151 | PNRC1 | na |  |  | 14528 | -0.496 | -0.0185 | No |
| 152 | PHLDA1 | na |  |  | 14990 | -0.561 | -0.0375 | No |
| 153 | B4GALT5 | na |  |  | 15298 | -0.617 | -0.0480 | No |
| 154 | DNAJB4 | na |  |  | 15495 | -0.649 | -0.0523 | No |
| 155 | PDE4B | na |  |  | 15522 | -0.656 | -0.0476 | No |
| 156 | IL7R | na |  |  | 15528 | -0.657 | -0.0418 | No |
| 157 | IFNGR2 | na |  |  | 15618 | -0.672 | -0.0402 | No |
| 158 | BTG1 | na |  |  | 15833 | -0.711 | -0.0449 | No |
| 159 | JAG1 | na |  |  | 15843 | -0.713 | -0.0388 | No |
| 160 | EIF1 | na |  |  | 15919 | -0.727 | -0.0360 | No |
| 161 | NFIL3 | na |  |  | 16230 | -0.795 | -0.0450 | No |
| 162 | TANK | na |  |  | 16289 | -0.808 | -0.0406 | No |
| 163 | RIPK2 | na |  |  | 16616 | -0.878 | -0.0496 | No |
| 164 | SLC2A3 | na |  |  | 16869 | -0.946 | -0.0541 | No |
| 165 | F2RL1 | na |  |  | 16911 | -0.960 | -0.0474 | No |
| 166 | BTG3 | na |  |  | 16989 | -0.983 | -0.0423 | No |
| 167 | TNFAIP8 | na |  |  | 17185 | -1.045 | -0.0429 | No |
| 168 | BIRC3 | na |  |  | 17468 | -1.140 | -0.0472 | No |
| 169 | GEM | na |  |  | 17475 | -1.142 | -0.0370 | No |
| 170 | PDLIM5 | na |  |  | 17595 | -1.194 | -0.0322 | No |
| 171 | NFE2L2 | na |  |  | 17653 | -1.221 | -0.0239 | No |
| 172 | LITAF | na |  |  | 17716 | -1.247 | -0.0157 | No |
| 173 | DDX58 | na |  |  | 18014 | -1.392 | -0.0184 | No |
| 174 | IFIT2 | na |  |  | 18078 | -1.429 | -0.0085 | No |
| 175 | AREG | na |  |  | 18122 | -1.456 | 0.0027 | No |
| 176 | NAMPT | na |  |  | 18342 | -1.596 | 0.0059 | No |
| 177 | IL6ST | na |  |  | 18809 | -2.032 | 0.0002 | No |
| 178 | CCRL2 | na |  |  | 18912 | -2.189 | 0.0150 | No |
Table: GSEA details [plain text format]

  

Fig 2: HALLMARK\_TNFA\_SIGNALING\_VIA\_NFKB      
 Blue-Pink O' Gram in the Space of the Analyzed GeneSet

  

Fig 3: HALLMARK\_TNFA\_SIGNALING\_VIA\_NFKB: Random ES distribution      
 Gene set null distribution of ES for **HALLMARK\_TNFA\_SIGNALING\_VIA\_NFKB**
